# The Effect of Gap Angle on Tensile Strength of Preceramic Base Metal Solder Joints

**Published:** 2015-12

**Authors:** Farnaz Fattahi, Zahra Hashemi Ardakani, Maryam Hashemi Ardakani

**Affiliations:** a Dept. of Prosthodontics, School of Dentistry, Shiraz University of Medical Science, Shiraz, Iran.; b Undergraduate Student, School of Dentistry, Shiraz University of Medical Science, Shiraz, Iran.

**Keywords:** Base Metal, Solder Gap angle, Solder Joint, Tensile Strength

## Abstract

**Statement of the Problem:**

Soldering is a process commonly used in fabricating dental prosthesis. Since most soldered prosthesis fail at the solder joints; the joint strength is of utmost importance.

**Purpose:**

The purpose of this study was to evaluate the effect of gap angle on the tensile strength of base metal solder joints.

**Materials and Method:**

A total number of 40 Ni-Cr samples were fabricated according to ADA/ISO 9693 specifications for tensile test. Samples were cut at the midpoint of the bar, and were placed at the considered angles by employing an explicitly designed device. They were divided into 4 groups regarding the gap angle; Group C (control group) with parallel gap on steady distance of 0.2mm, Group 1: 10°, Group 2: 20°, and Group3: 30° gap angles. When soldered, the specimens were all tested for tensile strength using a universal testing machine at a cross-head speed of 0.5 mm/min with a preload of 10N. Kruskal-Wallis H test was used to compare tensile strength among the groups (*p*< 0.05).

**Results:**

The mean tensile strength values obtained from the study groups were respectively 307.84, 391.50, 365.18, and 368.86 MPa. The tensile strength was not statistically different among the four groups in general (*p*≤ 0.490).

**Conclusion:**

Making the gap angular at the solder joints and the subsequent unsteady increase of the gap distance would not change the tensile strength of the joint.

## Introduction

Soldering is among the oldest methods used to connect the parts of fixed partial dentures by use of a metal.[1] In order to verify the metal framework seated properly on the abutment, the frame should be fit in the patient’s mouth. If the complex is imbalanced and shows rocking, it would be cut away and soldered.[2] The long-span bridges are cast in two sections and then the parts are soldered. In this case, the functional life of the soldered prosthesis depends on the mechanical properties of the soldering site.[3-5] The soldering site of a bridge is said to be the concentrating point for the stresses, especially tensile stresses.[6] Therefore, this joint must be strong enough to withstand the intraoral forces. The strength of the soldering site is influenced by several factors such as the alloy composition, soldering material, alloy contamination, indexing method, and material, wetting angle, as well as the shape and dimension of joint area.[7-10] One of the variables associated with the soldering strength is the gap distance; which has been evaluated in several studies and various results have been reported.[2, 4, 11-14] This gap was reported to be ideal within the range of 0.1 to 0.7mm. In most of these studies, the soldering point was cut at a steady distance and almost parallel. If the bridge is cast in two sections from the very beginning, the shape and distance of the solder site can be desirably determined and a steady gap will be included. Meanwhile, there are cases in which the bridge is cast in one section and it should be cut and soldered since it is imbalanced or some rocks on abutments are present. In that case, even if the soldering area is cut steadily on the desired distance by use of the considered disk, the gap would no longer have the desired shape and distance. It would often turn into two unparalleled surfaces that converge on one side and diverge on the other; also the desired distance might be only obtained on one side of the gap.

Depending on the rock intensity, the degree of angle created in the gap after cutting is so variable that soldering over some angles might hardly result in favorable strength. The current study was enrolled to evaluate the solder joint strength in conditions similar to the real clinical conditions in which the gap distance was different in various areas and the two surfaces were positioned angular. The null hypothesis was that increasing the gap angle would not change the tensile strength of the joint. 

## Materials and Method

**Figure 1:**
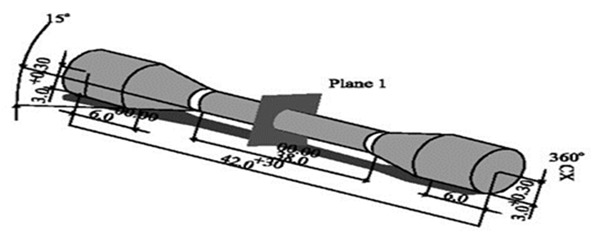
Bar dimensions

**Figure 2:**
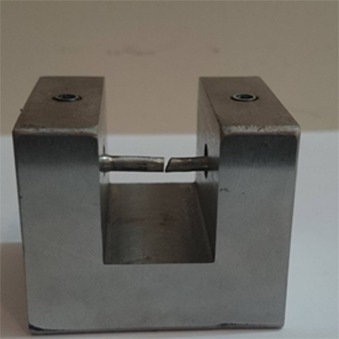
Custom device to hold the samples (lateral view)

A brass model with a 3mm-diameter bar was prepared according to ADA/ISO 9693 specifications for tensile strength test (Figure 1); 

The model had to be cut and soldered in the middle. Silicon impression was taken (Speedex; Coltene, Germany) from the model, and 40 wax models were prepared to fabricate 40 samples. Spruing and investing (Ernst Hinrichs; GMbH, Goslar, Germany) were done; as well as casting (Ni-Cr alloy; Wilhelm, Schutz, Germany) that was performed by use of an inducting casting machine (Dentsply; Germany) based on the manufacturer’s instructions. Carborundum disc was used to cut the sprues; the samples were finished and polished. Those samples which showed any deformation or any bubbles after casting were excluded from the study. A custom device was designed and fabricated to hold the specimens firmly while being positioned at the desired gap angle (Figure 2).

**Figure 3:**
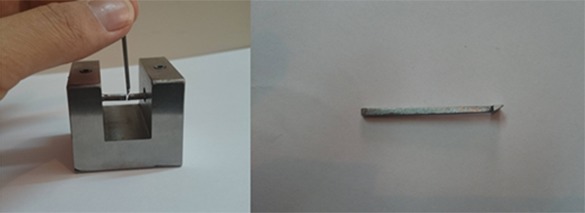
Metallic index to determine the gap angle

Four metal indices were designed and fabricated to position the solder joints at the desired gap angle (Figure 3). One of them was parallel sided with 0.2mm thickness and others were wedge shaped with different thickness at the base and at the top.

At the base, all of these three indices had the same thickness of 0.2mm, and at the top, they had different thicknesses of 0.7 mm, 1.3 mm, and 1.9 mm. The samples which had any bubbles at the cutting point were excluded. By using a 0.3mm carborundum disc, the specimens were cut at the midpoint by a nonstop cutting machine; then the metal indices were placed to determine the position and angle of joints to be soldered. The samples were divided into four groups regarding the solder gap angles; Group C: parallel gap with 0.2mm gap distance, Group1: 10° angle with 0.2 mm gap distance at the base and 0.7 mm at the top, Group2: 20° gap angle with 0.2mm gap distance at the base and 1.3mm at the top, Group3: 30° gap angle with 0.2mm gap distance at the base and 1.9mm at the top of the gap. 

The upper side of the gap on samples was marked with a groove. Soldering procedure was carried out with respect to Rosentiel’s recommended method.[15] Pattern resin (GC Corporation; Tokyo, Japan) was used to fix the samples in the desired positions while being invested and soldered. Resin was burned out by a torch at 300°C. Samples were subjected to a flux (High-fusing Bondal^TM^ Flux; Ivoclar Vivadent, Liechtenstein) and were then put in the furnace. Temperature was gradually raised up to 850°C until the samples turned red. At that moment, they were retrieved from the furnace. A pinpoint flame of gas-oxygen torch was used at its reducing zone concentrated on the joint space, and the solder was placed above the gap. The samples were placed in such a way that the wider opening of the gap was upward. After bench cooling for about 5 min, the assembly was ready to break the investment and evaluate the solders. The samples were tested by hand force. Joint irregularities were ground away and the subsequent finishing and polishing procedures was done.

The specimens were tested for tensile strength by a universal testing machine (Instron; Zwick 20, Germany) at a cross-head speed of 0.5 mm/min with a preload of 10N. After that, the specimens were examined concerning the location of failure. The collected data were analyzed by using SPSS software, version 17 (Chicago; IL, USA). Data were described by using mean±SD and minimum and maximum values. Kruskal-Wallis H test was used to compare the tensile strength among the groups. *p*< 0.05 was considered significant.

## Results

As demonstrated in Table 1, the mean tensile strength values in groups C, 1, 2 and 3 were respectively 307.84, 391.50, 365.18, and 368.86 MPa. Group 1 with 10° angle had the highest and group C with parallel gap had the lowest tensile strength.

**Table 1:** Comparison of the mean tensile strength (MPa) between the study groups.

**Angle**	**N**	**Min.**	**Max.**	**Mean ± S.D**	P^*^
0°	10	307.70	459.60	307.84 ± 148.16	0.490
10°	10	352.69	423.51	391.50 ± 30.66	
20°	10	325.77	430.59	365.18 ± 41.45	
30°	10	307.36	417.84	368.86 ± 43.95	

* Kruskal–Wallis H test

The tensile strength was not statistically different among the four groups in general (*p*≤ 0.490).

## Discussion

Long-span fixed partial prostheses usually fracture in clinical conditions when exposed to forces; thus, tensile test is considered appropriate for evaluation of the solder strength.[16] This study assessed the effect of gap angle on the tensile strength of joint.

Several studies evaluated the effect of gap distance on the joint strength and obtained various results.[4, 14, 17-18] In majority of the studies,[16, 18-19] the soldering surfaces were cut in the joint with a steady distance and the two surfaces were positioned parallel. However, in clinical conditions, if soldering is used to eliminate the rock of the bridge, the gap would not be parallel after cutting the site; the surfaces would be angular. The current study evaluated the three angles (10°, 20° and 30°) that cause unsteady increase of the gap distance and their effect on the tensile strength of the joint in comparison with the parallel state.

The null hypothesis was rejected and this study demonstrated that the angularity of the soldering site, increasing the angle, and the subsequent increase in the gap distance- although unsteady- would improve the joint strength compared with the state in which the surfaces are parallel. However, this difference was not statically significant. This can be due to the fact that penetration of the solder into the gap would be facilitated following the increase of the gap distance. Therefore, the probability of the presence of porosity in the connecting point would decrease and consequently the strength would be greater. Ryge[17] reported that reducing the gap distance down to 0.12mm would increase the porosity and decline the joint strength. In a study by Anwar *et al.*, the joint strength was reported to be higher at 0.5mm than 0.3mm; the reason was mentioned to be the increased possibility of generating porosity in the gap site following the decrease of the gap distance.[20] Likewise, Stade *et al.*[14] showed that increasing the gap distance would lead to enhanced joint strength. In that study, all samples failed at the joint point, indicating this point as the weakest point in this complex.

The current study revealed that, the considerable increase of the cutting angle up to 30° in addition to unsteady increase of the distance up to 1.9 mm would not have adverse effects on the joint tensile strength; however, the tensile strength is not the only required factor for the soldered bridge to be acceptable. Hence, the effects of this angle and the distance on the precision of the bridge complex including mesiodistal width of the bridge as well as the precision of the margin retainers must also be scrutinized in future studies. 

## Conclusion

This study evaluated the effect of gap angle on the tensile strength of preceramic base metal Nickel-Chromium solder joints. It can be concluded the angularity of the gap in solder joint and the subsequent unsteady increase in gap distance would not change the tensile strength of the joint significantly in comparison with parallel gap. Fraction of all samples at the solder joint space suggests this point as the weakest point of soldered assembly. 
